# Activation of Mirror Neuron Regions Is Altered in Developmental Coordination Disorder (DCD)–Neurophysiological Evidence Using an Action Observation Paradigm

**DOI:** 10.3389/fnhum.2019.00232

**Published:** 2019-07-11

**Authors:** Jessica M. Lust, Hein T. van Schie, Peter H. Wilson, Jurjen van der Helden, Ben Pelzer, Bert Steenbergen

**Affiliations:** ^1^Behavioural Science Institute (BSI), Radboud University, Nijmegen, Netherlands; ^2^Centre for Disability and Development Research (CeDDR), School of Behavioural and Health Sciences, Australian Catholic University, Melbourne, VIC, Australia

**Keywords:** developmental coordination disorder (DCD), MNS, mu desynchronization, EEG coherence, sequence learning, internal modeling

## Abstract

Children with Developmental Coordination Disorder (DCD) have difficulty performing and learning motor skills. Automatic activation of the mirror neuron system (MNS) during action observation and its coupling to the motor output system are important neurophysiological processes that underpin observational motor learning. In the present study, we tested the hypothesis that MNS function is disrupted in children with DCD by using sensitive electroencephalography (EEG)-based measures of MNS activation during action observation. Specifically, we predicted reduced mu-suppression and coherence in DCD compared with typically developing children. Neural activation of the motor network was measured by EEG, specifically event-related desynchronization (ERD) of mu rhythms and fronto-parietal coherence. Children (15 DCD/15 controls) were tested under two task conditions: observational learning (imitation of an observed action) and detection (report a deviant movement after observation). EEG-metrics were compared between groups using linear mixed-effects models. As predicted, children with DCD showed lower levels of mu suppression and reduced modulation of coherence during the observational learning task compared with their non-DCD peers. Notably, mu suppression was reduced in DCD over the entire imitation task (repetitions, and both observation and pause intervals). Action observation can be used for the acquisition of new motor skills. This form of learning entails the transposition of the observed action to the existing internal representations of the observer’s own motor system. The present neurophysiological results suggest that this process of learning is impaired in children with DCD. The results are discussed in relation to current hypotheses on mechanisms of DCD.

## Introduction

Developmental coordination disorder (DCD; American Psychiatric Association, 2013) is a common motor problem affecting 5%–6% of school-aged children (Zwicker et al., 2012). DCD significantly interferes with activities of daily living and school performance. Moreover, physical and psychosocial concerns related to DCD have been well documented (Zwicker et al., 2012, 2013, 2018; Harris et al., 2015; Karras et al., 2019). DCD is more common in boys than in girls and is often associated with psychopathology, particularly with attention-deficit/hyperactivity disorder (ADHD) and Autism Spectrum Disorders (ASDs)/autistic-type problems (Harris et al., 2015). The cause of DCD is largely unknown, but the motor difficulties do not result from major neurologic impairment or low intelligence (American Psychiatric Association, 2013). Experimental studies conducted over the past 20 years have shown that children with DCD have disruptions in motor control and in learning new motor skills through repeated practice (Wilson et al., 2013, 2017b). The studies in DCD have found evidence for motor control problems in various behaviors such as reaching, gait, posture, eye movement control, and imagined action (Wilson et al., 2003; Adams et al., 2014, 2017a; Smits-Engelsman et al., 2018). Notwithstanding this evidence, the cause(s) of DCD is unclear and remains a topic of current debate (Werner et al., 2012). One recent hypothesis that may account for the cluster of motor difficulties seen in DCD is an impairment of the mirror neuron system (MNS; Werner et al., 2012; Reynolds et al., 2015b; Wilson et al., 2017a,b).

The MNS consists of a distributed neural network of high-level sensorimotor regions in frontal and parietal cortices that contributes to humans ability to imitate and learn new behaviors through observation (Rizzolatti and Luppino, 2001; Buccino et al., 2004; Rizzolatti and Craighero, 2004; Iacoboni, 2005). A defining feature of mirror neurons is that they fire during the execution of a goal-directed action (e.g., grasping of an object), as well as during observation of a similar goal-directed action (Rizzolatti et al., 1996). Mirror neurons support the coupling of perception and action, and internal models that enable monitoring and control of executed actions (Miall, 2003; Kilner et al., 2007; Iacoboni, 2009). By their activation in response to seeing others’ actions (Buccino et al., 2001; Rizzolatti et al., 2001; Kohler et al., 2002; Maeda et al., 2002; Uddin et al., 2005), mirror neurons provide the observer with a direct sensorimotor perspective of the observed action including simulation of other peoples’ embodied experiences (Keysers and Gazzola, 2014) like the perception of touch (Keysers et al., 2004) and facial emotional expression (Jabbi and Keysers, 2008; Keysers and Gazzola, 2009).

The process by which action observation can elicit automatic activation of sensorimotor neural networks (like the MNS) is also known as *motor resonance*. Motor resonance occurs at the level of motor acts (Rizzolatti et al., 2001; Fogassi et al., 2005), that is the consecutive movements (e.g., reaching, grasping, turning, lifting) that together make up a motor action (e.g., opening and drinking from a bottle; Fogassi et al., 2005). In order to imitate correctly, the observer is required to execute individual motor acts in the correct sequential order and in reference to the desired goal (e.g., turn the cap instead of the bottle). Frontal and parietal regions of the MNS are believed to represent motor acts and action goals (Fogassi et al., 2005; Hamilton and Grafton, 2008; van Elk et al., 2014) and mirror neurons play an important role in linking individual motor acts in an action chain (Werner et al., 2012).

In line with the MNS hypothesis of DCD, systematic reviews of neuroimaging studies in DCD have shown hypo-activation in cortical and subcortical structures supporting motor function, including areas that overlap with the MNS (Biotteau et al., 2016; Wilson et al., 2017b). However, few recent studies have tested the MNS hypothesis of DCD by investigating brain activation during action observation and imitation tasks. Reynolds et al. (2015a) found reduced activation in the right inferior frontal gyrus (IFG) in DCD during action observation of finger sequencing compared with controls. The IFG is rich in mirror neurons and is activated reliably during action observation and imitation (Iacoboni and Dapretto, 2006). Furthermore, a region of interest (ROI) analysis in the same study of Reynolds et al. (2015a) found reduced activation of the IFG region during imitation in the DCD group, relative to controls, supporting the MNS hypothesis of DCD. A second functional magnetic resonance imaging (fMRI) study from the same group (Licari et al., 2015) corroborated these first findings by reporting reduced activation of the left IFG in DCD relative to controls using the same finger sequencing task as Reynolds et al. (2015a). A third fMRI study (Reynolds et al., 2019), however, failed to confirm the MNS hypothesis. Using a simple tapping task, reduced activation in DCD was only evident for structures outside the MNS.

To date, the MNS hypothesis of DCD has been investigated by fMRI, using relatively simple imitation tasks such as repetitive finger sequencing and finger tapping. Such tasks may not be representative of the motor learning and control difficulties that characterize DCD. In the current study, we selected electroencephalography (EEG) as it provides much more freedom to investigate complex movement sequences in the context of action observation. Furthermore, coherence measures in EEG provide a direct correlate of coupling in the MNS (i.e., the chaining of motor acts and coupling between action goals and means) which is fundamental to observational learning (van der Helden et al., 2010).

Current EEG-based methods show considerable promise in probing the integrity of the MNS. EEG studies in the motor domain have found the mu rhythm—oscillations around 10 Hz over sensorimotor regions surrounding the central sulcus—to desynchronize during motor execution (Pfurtscheller et al., 2006), motor imagery (Pfurtscheller and Neuper, 1997; Gonzalez-Rosa et al., 2015) and action observation (Cochin et al., 1999; Muthukumaraswamy et al., 2004; Lepage and Théoret, 2006; Braadbaart et al., 2013). These findings indicate that *mu desynchronization* may be used as a measure of motor resonance reflecting activation of low level sensorimotor structures (e.g., primary and secondary sensory and motor regions; Pfurtscheller and Neuper, 1997; Hobson and Bishop, 2017). More precisely, mu desynchronization is the downstream effect of activation in higher-level MNS regions [i.e., inferior parietal lobe (IPL) and IFG] which influence sensorimotor activation during action observation or motor execution (Hobson and Bishop, 2017). Importantly, mu desynchronization has been observed during the process of observational learning (van der Helden et al., 2010); these EEG data suggest that sensorimotor representations are activated during action observation for subsequent imitation and that performers use motor imagery to help rehearse new action sequences following their presentation.

A more direct electrophysiological correlate of mirror neuron activity—*mu coherence—*is suggested by van der Helden et al. (2010). In their study on observational learning, they found that coherence in mu frequency—reflecting functional coupling between frontal and parietal motor regions—increased when participants watched action sequences for subsequent imitation. Functional coupling between frontal and parietal regions of the MNS is thought to reflect the linking of action means (e.g., choice of hand) and action goals (choice of target location) in imitation. Intriguingly, participants with stronger frontoparietal mu coherence were more accurate in their imitation performance which supports the view that frontoparietal coherence provides a direct correlate of activation of MNS regions during observational learning.

**Figure 1:**
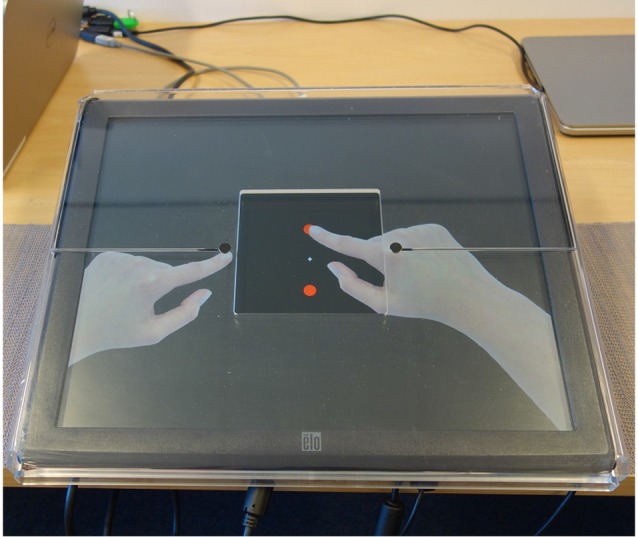
Example of a pointing frame in which the index finger of the right hand is moved to the upper (red) target dot. The index finger of the left hand is at the start location (one of the two black response buttons). For each pointing frame, only one of the two hands moved to one of the target dots.

In the present study, the paradigm of van der Helden et al. (2010) was used to provide a test of the MNS hypothesis of DCD. Here, participants learn new action sequences through observation in order to imitate them. The action sequence consists of four pointing movements with the left and the right hand to one of two target locations, sagittally aligned between both hands (see Figure 1). Each sequence was repeated three times to permit observational learning. Between each sequence, a 5 s pause was presented. A control condition presented the exact same stimuli but with the instruction to just observe and detect an occasional oddball stimulus (one of the hands presented in grayscale instead of in color).

In accordance with the hypothesized disruption of the MNS in DCD, we predicted, first, that children with DCD will show reduced mu desynchronization (in terms of mu power) during observational learning, relative to matched controls (i.e., typically developing children). Second, we predicted that frontoparietal coherence in mu frequency during action observation would be reduced in DCD relative to controls. On a behavioral level, we expected control children to outperform the children with DCD in imitation accuracy.

The results of this study will add to our understanding of the neurophysiological causes of DCD impairments and will help understand the deficits in motor performance and learning in light of current functional models of observational learning and imitation. These insights will inform existing treatment programs.

## Materials and Methods

### Participants

Thirty children participated in the study (15 diagnosed with DCD, 15 Controls; details shown in Table 1). Children with DCD were recruited from three sources: (i) a mainstream secondary school that also has facilities for children with special physical needs; (ii) a Dutch association for parents of children with neurodevelopmental problems like DCD; and (iii) a database of children that participated in an earlier project from our research group. Typically developing (control) children were invited through the same secondary school or *via* our database. Children’s parents gave written informed consent and children approved verbally in accordance with the Declaration of Helsinki. The study was approved by the ethics committee of the Faculty of Social Sciences at Radboud University (ECSW2014-1006-223).

**Table 1:** Summary of participant characteristics (*N* = 30).

	Sex Boys/Girls	Age (years) Min-max (*M*; SD)	M-ABC2Total Min-Max (*M*; SD)	Writing hand Right/Left	Education Main/Special	Diagnosed with AD(H)D
DCD	14/1	9.1–13.8 (11.1; 1.6)	0.1–5.0 (1.27; 1.7)	13/2	12/3	2*
Controls	12/3	9.5–13.7 (11.3; 1.6)	16–91 (56.79; 22.8)	14/1	15/0	0

Note. *As indicated by parents. None of these children scored within the clinical range of the ADHD questionnaire (AVL; Scholte and Van der Ploeg, 2004). They did not use any medication.

Having a formal DCD diagnosis was an inclusion criterion for the DCD group. All children (aged 9–13 years) in the DCD group had already been clinically diagnosed with DCD (DSM Criterion C). Parents sent in a copy of the medical file stating this diagnosis. If the assessment was longer than 1 year prior, children were re-assessed on the Movement Assessment Battery for Children-2 (M-ABC-2; Dutch translation—Smits-Engelsman, 2010). The inclusion cut-off on the M-ABC-2 was a Total score at or below the 5th percentile (DSM Criterion A; Table 1). In addition, the Dutch version of the Developmental Coordination Disorder Questionnaire (DCD-Q) was completed by all parents (CVO—Schoemaker et al., 2006) as a measure of their child’s motor performance relative to peers. All children in the DCD group scored in the DCD range on this questionnaire. As well, all parents reported that their child had significant motor difficulties that interfered with everyday functioning and that required treatment (Criterion B).

All children in the control group were tested with the M-ABC-2 and their parents filled in the DCD-Q. Inclusion criterion for controls was a M-ABC-2 Total score above the 15th percentile (Table 1) and no indication for DCD based on the DCD-Q. Control children were matched on age and sex to the DCD-group.

Attendance of mainstream education classes was taken to indicate an IQ-score within normal range. Three children in the DCD group attended special education. Their Total IQ scores were 75, 80, 96 as reported by their parents from file. None of the children had any neurological or visual problems, as reported by parents (Criterion D).

### Apparatus

Custom software, developed in Presentation (Neurobehavioral Systems, Albany, NY, USA) was used to present the tasks and record data. Children sat in front of a 19-inch touch-screen (ELO 1928L Desktop Touchmonitor) that was tilted at 10° towards them (Figure 1). Their arms rested comfortably next to the touch-screen, and their feet were placed flat on the ground. A transparent hardcase cover with a 12 × 12 cm open space in the center was placed over the touch-screen. Two black response buttons (1 cm diameter) were mounted on the cover at the edges of the open space (Figure 1). Hand stimuli were rendered from a full colored photo of one of the experimenter’s pointing hands (JML), mirrored to create a left and a right hand of identical form.

### Procedure

At the start of each block of trials, children were informed on-screen if the block required imitation or just detection. For an imitation block, the text “Look at the movements to imitate them later” was presented. For a detection block, “See if a gray hand is presented” was presented.

At the beginning of each trial (of either task), the child was presented with the text “fingers off the buttons,” displayed until they released fingers from both response buttons. The child was instructed by the researcher to place their hands adjacent to the touch screen apparatus, one hand to each side. Subsequently, a start frame was presented for 2 s showing a fixation cross in the center of the screen, two red target dots (1 cm diameter) presented 3 cm above and below the fixation cross, and left and right hands with index fingers pointing at the location of the two response buttons. Next, the children were presented with a sequence of four pointing movements on the screen. Movements were shown by presenting pictures of hands at different locations (for a similar method see van der Helden et al., 2010). Each movement consisted of the start frame presented for 500 ms and a pointing frame of 500 ms in which the left hand or the right-hand stimulus was displaced so that the tip of the index finger was positioned at one of the red target dots (see Figure 1). Four different pointing frames were possible depending on the hand (left, right) and the target location (up, down) to which the hand pointed. A sequence consisted of four consecutive movements. The presentation of these successive frames resulted in the visual impression of the hands going sequentially back and forth between their starting position and one of the two target dots, for a period of 4 s.

The order of movement sequences was pseudo-randomized. However, over successive sequences, we ensured that the same target location was never visited four times in a row. Similarly, sequences with four consecutive pointing movements towards the same target location were precluded. With these constraints, 26 different orders were possible. Each block consisted of 19 randomly-selected trials (from the 26).

After the 4 s presentation of the first movement sequence, a 5,000 ms pause interval was presented. During the pause, the location dots were presented in white (instead of red). In each trial, the sequence and the consecutive pause interval were presented three times to allow memorization. During observation of the movement sequences and pause intervals, participants sat with their hands resting adjacent to each side of the screen.

#### Imitation Blocks

In imitation blocks, participants were instructed to imitate the movement sequence after the third pause interval. The text “fingers on the buttons” was presented and when both fingers were in the correct position on the buttons, the text “start imitation” was presented. Participants could start imitating whenever they were ready. At the completion of all four consecutive movements, participants received text feedback about their imitation accuracy (“Answer is right” or “Answer is incorrect”).

#### Detection Blocks

In detection blocks, participants were instructed to watch the movement sequence and to detect an oddball stimulus, i.e., a hand presented in grayscale instead of full color. In each block, 4 of the 19 trials were programmed to contain an oddball. The trials in which the oddballs were presented were selected randomly, as was the ordered position of the oddball within a trial. After the three sequence repetitions, the text “have you seen a gray hand?” was presented on the screen, followed by the presentation of “yes” next one button and “no” next to the other button. The assignment of “yes” and “no” to the left or to the right button was chosen randomly in each trial to prevent response preparation before the onset of the question. Participants received feedback whether their answer was correct or not, similar to the imitation block.

#### Counter-Balancing

The order of tasks was counterbalanced based on participant number (even/odd). In total two imitation blocks and two detection blocks were presented in an ABAB design. Three practice trials were completed before the first block of each task. Each block consisted of 19 trials that were presented in three sub-blocks of six, six, and seven trials respectively. A short break was taken between each sub-block.

### Electrophysiological Recording

A 32-channel active electrode system (actiCapMedCat B.V. Netherlands) was used to record the EEG. Signals were amplified by a 32-channel BrainAmp EEG amplifier. Electrodes were fixed to an electrode cap of appropriate size, according to the international 10-20 system at AFz, Fz, FCZ, Cz, Pz, Oz, Fp1/2, F3/4/8/7, FC1/2/5/6, C3/4, T7/8, CP1/2/5/6, P3/4/7/8, Oz, O1/2, PO9 (Pivik et al., 1993; American Electroencephalographic Society, 1994). A ground electrode was placed on the upper part of the sternum. The left mastoid served as an online reference. Electrode impedances were kept below 25 kOhm. EEG was sampled at 500 Hz with 250 Hz low pass filter and a 50 Hz notch filter.

Electro-oculography (EOG) was recorded from bipolar electrodes on the outer canthi of each eye (horizontal EOG) and above and below the right eye (vertical EOG).

Electromyograms (EMGs) were recorded to rule out the possibility of covert muscle activity using bipolar electrodes on both forearms over the extensor digitorum. Reference electrodes were placed just above the elbows. The EMG was closely monitored online by the experimenter.

### Data Analysis

For each participant, the recorded EEG and EMG data were analyzed offline using BrainVision Analyzer (BVA, Version 2.1.1.2516 Brain Products GmbH, Germany).

#### EEG

EEG data were re-referenced offline to linked mastoids and filtered using a 4–14 Hz (order 8) zero shift Butterworth filter and a 50 Hz notch filter. Ocular correction was performed using the Independent Component Analysis (ICA) procedure. Artifacts were discarded using an automated procedure (maximal allowed voltage step: 50 μV/ms; maximal allowed difference of values in intervals: 200 μV; minimal allowed amplitude: −50 μV; maximal allowed amplitude: 50 μV; lowest allowed activity in intervals: 0.5 μV; 220 ms before and after the event were marked as bad). This resulted in an average of 1.2% (SD = 1.08) discarded data per participant for the imitation and detection task.

Trials with an oddball stimulus and trials with false alarm (i.e., trails where subject incorrectly indicated that they saw the oddball) in the detection task were excluded from the EEG analyses to rule out the possibility of contamination by response preparation (van der Helden et al., 2010).

#### EMG

The recorded EMG signals were filtered offline with a band pass of 30 Hz to 70 Hz and rectified. Fast Fourier Transformations (FFTs) were run on the EMG segments (512 points, Hanning window of 10%) and averaged to create separate power frequency spectra for imitation and the detection task.

### Analysis of Mu Power

FFT were run on the artifact-free EEG segments (512 points, Hanning window of 10%) and averaged to create separate power frequency spectra for the observation, pause and execution intervals for each repetition of the imitation and detection tasks.

Following van der Helden et al. (2010), each child’s individual mu rhythm band was determined by subtracting the power frequency spectrum of the execution phase for the imitation task (which contained the lowest mu activation due to hand movements) from the power frequency spectrum for the observation phase in the detection task. For each individual, maximal mu modulation within the 7.5–12.5 Hz frequency range was identified to determine each individual’s mu peak frequency. Mu power was calculated using a ±1.0 Hz frequency bandwidth around each individual’s mu peak frequency. Mu modulation at sensorimotor regions was analyzed based on the pooled data from electrodes FC1, FC2, C3, C4, CP1, and CP2 (see Drew et al., 2014).

### Analysis of Mu Coherence

Current source density (CSD) analysis was applied (order of splines = 4, 10 polynomials) on the artifacts-free EEG segments. Subsequently, an FFT (512 points, Hanning window of 10%) was run and coherence values were calculated for all channel pairs using the magnitude-squared coherence procedure. Average coherence across all electrode pairs and tasks was calculated per participant to determine individual peak coherence frequencies, i.e., the frequency with maximum coherence in the 7.5–12.5 Hz frequency range. Individual coherence values were calculated by taking the average coherence activation in the ±1.0 Hz band around each individual’s coherence peak frequency.

To identify the networks that are responsive to the observation of movements, coherence averages for the observation interval were subtracted from coherence averages for the pause interval during which no hand movements were presented (van der Helden et al., 2010). These coherence difference scores were transformed to *z*-values to identify electrode pairs with the strongest coherence during the observation of movement (*z*-values < −1). Average coherence in this observation network was used for statistical analysis.

### Analysis of EMG Data

No systematic muscle activity was detectable during the observation and pause periods of the tasks. EMG data were analyzed with linear mixed effects models, using the log_10_ normalized EMG power data. Fixed effects were Task (detection/imitation), Group (DCD/Controls), as well as the interaction-effect. Subject was entered as a random factor. Average EMG power in the imitation task was 0.08 μV (SD = 0.04) and 0.06 μV (SD = 0.04) in detection task. The linear mixed effects model on the log_10_ normalized EMG data showed a main effect of task, where the estimate for the mean (log_10_ transformed) EMG power in the imitation task is 0.08 (*p* < 0.001) higher than the estimate for the overall mean (intercept). There was no main effect of group (Estimate = −0.001, *p* = 0.979) nor a significant group by task interaction effect (Estimate = 0.01, *p* = 0.482). These findings suggest that children may have moved slightly more during the imitation task than during the detection task. The absence of group and interaction effects indicate that any group effects on EEG metrics (mu or coherence) cannot be explained by muscle activation factors.

### Statistical Analyses

Group differences in the number of errors in the imitation and detection tasks were analyzed with a binomial two-level regression model with a random subject effect.

The distribution of Mu power at sensorimotor regions deviated from normality. A log_10_ transformation was applied to obtain more normally distributed data.

Movement-related suppression of mu rhythms was analyzed with linear mixed effects models, using the log_10_ normalized mu power data. Correlations between the 12 observations per child showed little variation, ranging from 0.88 to 0.98. To account for these correlations, subject was entered as a random factor in the mixed effects model. The resulting intraclass correlation (between two particular observations from the same child) was 0.94. Fixed effects were Task (detection/imitation), Period (Pause/Observation), Repetition (1–3) and Group (DCD/controls), as well as their possible interaction-effects.

A log_10_ transformation was also applied to the average coherence values in the observation network during observational learning. These data were then also analyzed using linear mixed effects models. Correlations between the six observations per child showed little variation, ranging from 0.84 to 0.97. To account for these correlations, subject was entered as a random factor. The resulting intraclass correlation (between two particular observations from the same child) was 0.82. Fixed effects were Period (Pause/Observation), Repetition (1–3) and Group (DCD/controls), as well as their possible interaction-effects.

Effect coding was used for all independent variables. Therefore, the main effects associated with factor levels represent deviations from the grand mean, which is given by the model’s intercept.

To arrive at a parsimonious but still good-fitting model, we used a backward procedure in which the least significant highest order interaction terms were eliminated from the full model if this did not result in a significant change in model fit.

All statistical analyses were performed in R (R Core Team, 2017) with package lme4 (Bates et al., 2015).

## Results

Data from two participants (one control, one DCD) were excluded from further analysis because excessive artifacts precluded a reliable estimate of coherence (a minimum of 30 valid segments per condition was used as a criterion). Analyses were performed on the remaining group of 14 controls and 14 children with DCD.

### Response Errors

**Figure 2:**
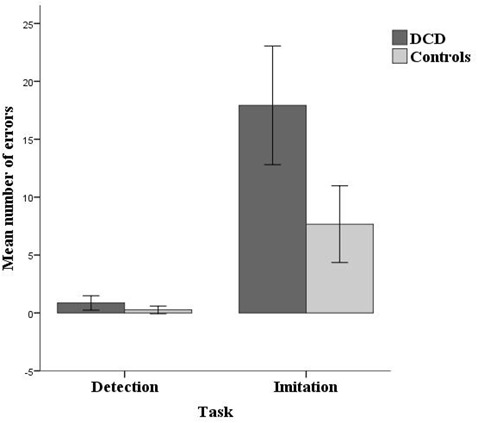
Mean number of errors per group (14 developmental coordination disorder (DCD)/14 Controls) and task (Detection/Imitation). Errors bars represent the 95% CI.

Response errors per group and task are displayed in Figure 2. Children with DCD made significantly more errors than controls, the estimate for the logit being 0.52, *z* = 2.35, *p* = 0.019. For both groups the number of errors was higher in the imitation task than in the detection task, logit estimate = 1.92, *z* = 12.19, *p* < 0.001. Analysis of error performance showed a no significant task by group interaction effect, logit estimate = 0.25, *z* = 1.59, *p* = 0.112.

### Mu Power

**Figure 3:**
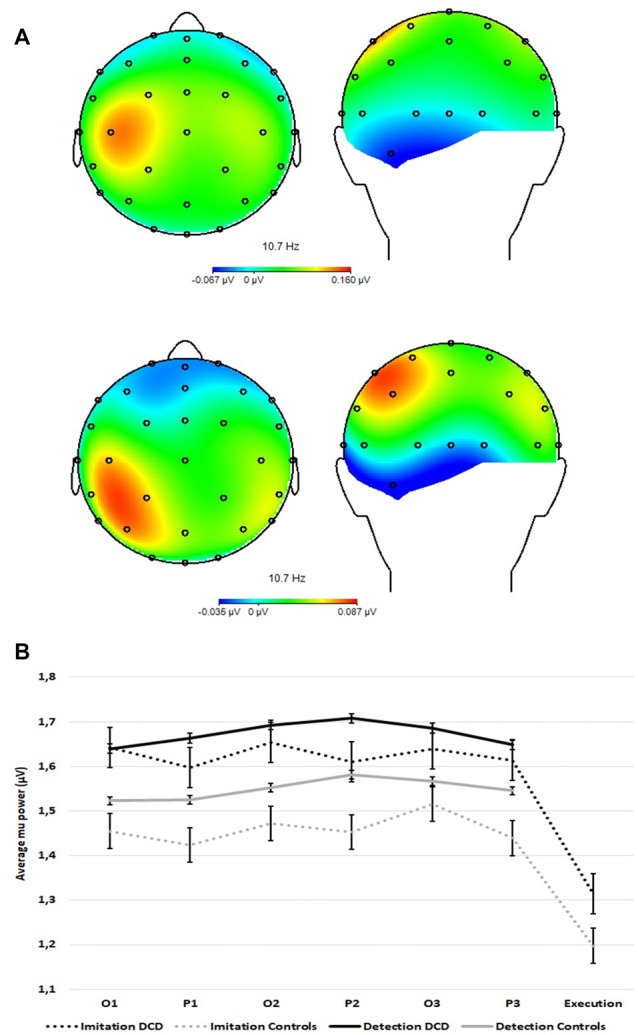
**(A)** Topographic representation of the maximal difference in averaged mu power over sensorimotor regions between the imitation task and the detection task for controls (n =14, top plots) and DCD (*n* = 14, bottom plots). **(B)** Averaged mu power over sensorimotor regions (FC1, FC2, C3, C4, CP1, and CP2) over time within trials as a function of task (imitation and detection) and group (14 controls and 14 DCD). O1 = first observation, P1 = first pause period, etc. Error bars represent standard errors (SE).

Figure 3A provides a topographic representation of the maximal mu power difference between the imitation task and the detection task for controls (top) and DCD (bottom). Figure 3B shows the modulation of the observed original (not log-transformed) averaged mu power over sensorimotor regions as a function of group (DCD, controls), task (imitation, detection), period (observation, pause) and repetition (1–3). All 4-way and 3-way interactions and the 2-way interaction terms with repetition could be eliminated without a significant decrease in model fit. The final model explained 17.89% of the within-subjects variance and 6.14% of the between-subject variance in the empty model.

Fixed effects of the final model are shown in Table 2. The mean for the DCD group (0.18 + 0.02 = 0.20) did not differ significantly from that of controls (0.18 − 0.02 = 0.16). The significant effect of task shows stronger mu-desynchronization in the imitation task than in the detection task, as expected. The significant task by group interaction revealed that for the DCD group the mu-desynchronization during observation and pause periods in the imitation task was less pronounced than for controls (the effect of task in the DCD group is −0.01 + 0.003 = −0.007 compared to −0.01 to 0.003 = −0.013 in the control group). In addition, the significant task by period interaction shows stronger mu-desynchronization during the pause intervals of the imitation task, compared with the detection task for both groups.

**Table 2:** Fixed effects of the final linear mixed effects model to estimate the log-transformed average mu power over sensorimotor regions.

	Estimate (SE)	*t*-value (df)	*p*-value
Intercept	0.18 (0.02)	9.60 (28)	<0.001
Group (DCD)	0.02 (0.02)	0.89 (28)	0.383
Task (imitation)	−0.01 (0.001)	−6.79 (308.01)	<0.001
Period (pause)	−0.003 (0.001)	−1.77 (308.01)	0.078
Repetition 2	0.003 (0.002)	1.62 (308.01)	0.106
Repetition 3	0.002 (0.002)	1.12 (308.01)	0.263
Period (pause) * Task (imitation)	−0.003 (0.001)	−2.25 (308.01)	0.025
Period (pause) * Group (DCD)	<0.001 (0.001)	0.03 (308.01)	0.979
Task (imitation) * Group (DCD)	0.003 (0.001)	2.28 (308.01)	0.023

Note: linear mixed model fit by maximum likelihood *t*-tests use Satterthwaite approximations to degrees of freedom. The estimate of repetition 1 equals −(−0.003 + 0.002) = −0.005. *Indicates the interaction term.

### Mu Coherence

**Figure 4:**
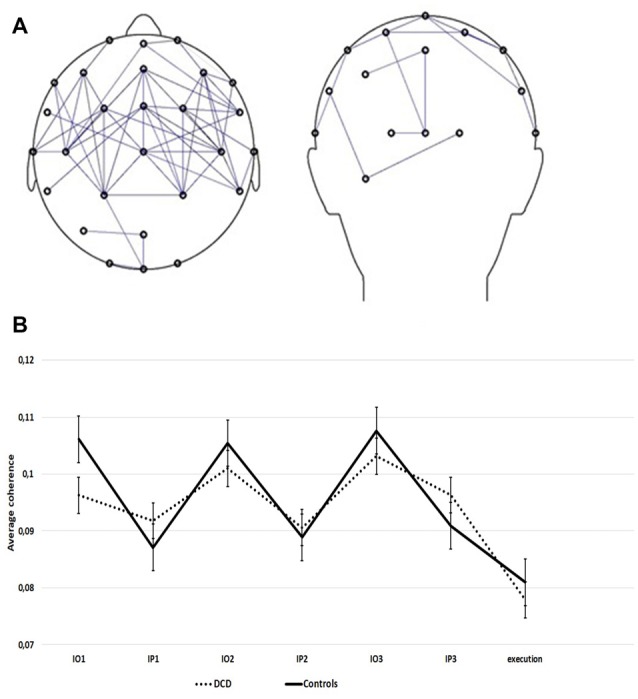
**(A)** Fronto-parietal coherence network. Pairs of electrodes showing higher mu-frequency coherence during the observation of movement sequences than during the pause interval. Only electrode pairs with the largest normalized coherence effect (*z* < −1) are shown. **(B)** Average coherence within the observation network (*z* < −1) during the imitation task, for the DCD and control group (14 controls, 14 DCD). Data are presented separately for the repeated observation of motor sequences (IO1, IO2, IO3) and consecutive pause intervals following each sequence (IP1, IP2, IP3). Error bars represent SE.

Figure 4A represents the observation network, a fronto-parietal coherence network that was found to be activated more strongly during the observation of movement sequences than during the pause intervals of the imitation task. Figure 4B presents the observed original (not log-transformed) average coherence within this network for the consecutive observation and pause intervals of the imitation task for DCD and controls. All 3-way interactions and the 2-way interaction terms with repetition could be eliminated without a significant decrease in model fit. The final model explained 42.0% of the within-subjects variance and 1.5% of the between-subject variance in the empty model. Fixed effects of the final model are shown in Table 3.

**Table 3:** Fixed effects of the final linear mixed effect model to estimate the log transformed average coherence in the observation network.

	Estimate (SE)	*t*-value (df)	*p*-value
Intercept	−1.02 (0.02)	−61.76 (28)	<0.001
Group (DCD)	<0.001 (0.02)	−0.005 (28)	0.996
Period (pause)	−0.03 (0.003)	−8.96 (140)	<0.001
Repetition 2	−0.002 (0.004)	−0.56 (140)	0.576
Repetition 3	0.01 (0.004)	2.31 (140)	0.023
Period (pause) * Group (DCD)	0.01 (0.003)	3.93 (140)	<0.001

Notes. Linear mixed model fit by maximum likelihood *t*-tests use Satterthwaite approximations to degrees of freedom. The estimate of repetition 1 equals −(−0.002 + 0.01) = −0.098. *Indicates the interaction term.

The mean coherence in the DCD group did not differ significantly from that in the control group, the estimate for the deviance of each group from the group mean is close to zero (Table 3). As expected, coherence in the observation network varied as a function of period. Coherence was stronger during the observation of movements than during the pause periods. This modulation was reduced for children with DCD as shown by the significant period * group interaction term. Separate analyses per group revealed that the estimate for period was significant in each group, but smaller within the DCD group [Estimate = −0.01 (SE = 0.004), *p* = 0.001] than in the control group [Estimate = −0.04 (SE = 0.004), *p* < 0.001]. Overall, coherence was strongest during the third repetition.

## Discussion

Our study investigated the MNS hypothesis of DCD which has been proposed as an explanation for the difficulties in learning and performing new motor skills in these children (Werner et al., 2012; Reynolds et al., 2015b; Wilson et al., 2017a,b). We tested the hypothesis by comparing children with and without DCD on EEG mu power and coherence during observational learning and imitation of movement sequences. The results supported the MNS hypothesis: first, mu suppression during observational learning was significantly reduced in children with DCD relative to typically developing children. Second, task-specific increases and decreases in mu coherence during observation and pause intervals of the imitation task were found to be significantly reduced in children with DCD, relative to the controls. The results of our study provide perhaps the first direct test and support of the MNS hypothesis of DCD using brain electrophysiology. These results corroborate recent fMRI studies that show preliminary evidence of atypical functioning of the MNS in DCD (Licari et al., 2015; Reynolds et al., 2015a; Wilson et al., 2017b).

Like earlier EEG studies on imitation (van der Helden et al., 2010; Braadbaart et al., 2013; Hobson and Bishop, 2017), mu power was suppressed during the perception of movements for subsequent imitation. Importantly, and in support of the MNS hypothesis in DCD, the significant Task by Group interaction (Figure 3B) shows reduced mu suppression in the DCD group over the entire imitation task (including all three repetitions, and both observation and pause intervals). This result indicates a prolonged reduction in mu suppression during the observation of actions with the intention to imitate, consistent with a general impairment in activating the MNS to levels shown in typically developing children under conditions of observational learning.

It is important to note that the reduction in mu suppression in the DCD group during the imitation task cannot be explained as an indirect effect of posterior alpha. In general, studies that investigate mu should be careful to exclude occipital alpha as a potential source of activation that may be picked up at central electrodes overlaying the sensorimotor strip (van der Helden et al., 2010; Hobson and Bishop, 2017). The topographical representation of mu suppression over the scalp (Figure 3A) confirms that mu suppression originates from bilateral central sources instead of posterior sources. Finally, analysis of alpha over posterior electrodes—similar to van der Helden et al. (2010) and not reported here—indicated large differences in the temporal profiles of mu and alpha, further highlighting their independence.

While earlier studies have consistently shown that mu is a direct correlate of activation of MNS regions (Arnstein et al., 2011; Braadbaart et al., 2013), we should nonetheless be cautious in drawing broad conclusions about MNS impairments. Mu is not linked uniquely to the MNS, but is also known to be activated when performing cognitive functions that are associated with motor activity such as language, empathy, motor imagery (Hobson and Bishop, 2017), and the observation and execution of intransitive actions (non-tool related) that rely on canonical neurons instead of mirror neurons (Muthukumaraswamy et al., 2004; Ulloa and Pineda, 2007; van Elk et al., 2008; Proverbio, 2012).

Group differences in the use of motor imagery may not explain group effects on EEG metrics observed here. Using a similar paradigm to ours, van der Helden et al. (2010) found that mu power was suppressed in the pause interval following movement observation, and interpreted this as the use of motor imagery in preparation of later imitation. In the current study, a similar effect was found in both groups, which suggests that children with DCD as well as controls engaged in motor imagery. Interestingly, as there was no significant interaction between group and period, our findings suggest that both DCD and non-DCD groups engaged in motor imagery to a similar extent. Taken together, performance differences in imitation between DCD and controls are not likely to be explained by differences in motor imagery in the current study.

In addition to mu suppression, the current study employed mu coherence as a measure of functional coupling within the MNS. Whereas mu suppression originates from primary somatosensory cortex (Salenius et al., 1997), and/or from areas surrounding the central sulcus (Miller et al., 2007), mu coherence is thought to reflect activation of MNS regions (van der Helden et al., 2010). We found a network of functional connectivity between lateral frontal and parietal electrodes comparable to van der Helden et al. (2010). Although no source localization was performed and no studies have yet investigated the exact neural foundation of this effect, it is likely that mu coherence during action observation reflects functional coupling in the MNS between action goals (i.e., the target location to which the hand points) and action means (i.e., the specific hand used for pointing), influencing the accuracy of imitation (see van der Helden et al., 2010). In line with the MNS hypothesis of DCD, mu coherence between frontal and parietal regions was found to vary more in control children than in DCD. More specifically, frontoparietal mu coherence in DCD was reduced during the observation of movement sequences; by comparison, mu coherence was somewhat higher during pause intervals (see Table 3, Figure 4). This suggests that children with DCD may have processed information about the subsequent movement goals and means differently in the consecutive parts of the imitation task. Put another way, information about movement goals and means was less well integrated in frontal and parietal regions of the MNS in DCD during action observation. In contrast, during pause intervals, functional coupling between frontal and parietal regions was somewhat stronger in the DCD group than in controls.

These findings suggest that during observation of movement sequences, children with DCD may find it more difficult to correctly couple sequential movement goals and movement means, and either attempt to compensate or to catch up during the pause intervals. Future research should investigate this hypothesis of reduced functional coupling and compensation during rest using the technique of thought listing; this technique can help isolate the point at which children with DCD experience difficulty and how they compensate during pause intervals.

In addition to motor simulation in response to the observation of goal-directed actions, our sequential observation learning task (van der Helden et al., 2010) probably also required executive functions such as working memory or cognitive control in order to remember consecutive hand-target combinations (see Buccino et al., 2004). As such our behavioral findings may reflect a combination of impairments in the MNS as well as decreased performance in executive functioning in DCD (Piek et al., 2004). Future EEG research may investigate the relative contribution of these functions as well as the integration between the MNS (reflected in mu power and coherence) and executive functions (reflected in theta power over frontal midline electrodes; Mitchell et al., 2008) in observational learning in DCD.

Together, results for mu power and mu coherence are consistent in suggesting an impairment of the MNS in DCD. It appears that children with DCD are less able to enlist the MNS for observational learning. As a consequence, accurate recognition of consecutive action goals and action means, and particularly their coupling, is disturbed during action observation. Although children with DCD appear to engage in motor imagery during pause intervals, this strategy is apparently insufficient to compensate for the initial impairment in action observation. As brain areas that constitute the human MNS are known to continue to develop throughout adolescence and beyond Kilner and Blakemore (2007) these capabilities might change as development progresses. Future studies should directly compare MNS function in children, adolescents and adults with and without DCD (preferably longitudinally) to study the development of MNS function in DCD.

An important clinical question is to what extent these findings can be translated into training procedures. Training of functions involving the MNS such as action observation, joint action, and imitation are recommended. More specifically, training could target compensatory functions such as motor imagery that are also involved in observational learning. In line with these suggestions, Adams et al. (2016) have developed a protocol to apply a combined action observation and motor imagery training to children with DCD. Pilot study results are positive (Wilson et al., 2016; Adams et al., 2017b).

Imitation is also fundamental to the development of social skills (Iacoboni and Dapretto, 2006). Neurons within the MNS have been found to not only code the observed motor act but also the intention of the observer (Fogassi et al., 2005). This has led to the hypothesis that dysfunction in the MNS might be a core deficit underlying deficits in social behavior like in ASD (Iacoboni and Dapretto, 2006). Compromised MNS function in DCD may, therefore, add to the debate concerning the possible overlap between the sensorimotor and cognitive problems of children with DCD and children with ASD (Williams et al., 2001; Caçola et al., 2017). An interesting question is whether a dysfunctional MNS in DCD is specific to motor learning or if other functions that rely on a well-functioning MNS might be affected as well. Such effects would add weight to the MNS hypothesis of DCD. In addition, investigating social and communicative functions in DCD might help to understand the functional differences in development that lead to DCD and ASD and the co-morbidity that may exist between them. Although various theories have proposed that MNS disruptions may provide a basis for specific disorders such as ASD (Iacoboni and Dapretto, 2006), DCD (Werner et al., 2012), psychopathy (Fecteau et al., 2008), schizophrenia (McCormick et al., 2012) and Alexithymia (Moriguchi et al., 2009), it is entirely unclear how deficits in MNS functioning might lead to such diverse disorders. This is an important question to address in future studies. DCD is interesting in this respect because the disorder is defined by deficits in the motor domain, in contrast to other disorders that are defined in terms of impairments in cognitive and socio-emotional domains such as language, theory of mind, and empathy.

## Conclusion

In conclusion, our results provide clear and direct evidence of MNS dysfunction in DCD as shown in behavior, mu power, and mu coherence. These findings corroborate previous fMRI studies that have shown some support for the MNS hypothesis of DCD. These results have important implications for how motor imagery and action observation can best be implemented in the treatment of DCD. As our study is the first to find electrophysiological evidence to support this hypothesis, additional research is needed to replicate our EEG findings, to determine the underlying neural source of mu coherence, and to further investigate the scope of difficulties in observational learning in DCD.

## Data Availability

The datasets for this manuscript are not publicly available because the raw data supporting the conclusions of this manuscript will be made available by the authors, without undue reservation, to any qualified researcher. Requests to access the datasets should be directed to j.lust@pwo.ru.nl or j.lust@bsi.ru.nl.

## Ethics Statement

The study was approved by the ethics committee of the Faculty of Social Sciences at Radboud University (ECSW2014-1006-223).

## Author Contributions

All authors have contributed to the work in a meaningful way. JL, HS and JH designed the experiment. JL conducted the experiment. JL and HS analyzed the data. BP and JL conducted the statistical analyses. JL, HS, BS and PW interpreted the results and wrote the manuscript. All authors have seen, reviewed and approved the manuscript.

## Conflict of Interest Statement

The authors declare that the research was conducted in the absence of any commercial or financial relationships that could be construed as a potential conflict of interest.
